# “Picking the right person … can make or break the whole deal”: Development of a layperson injector selection tool for administration of home-based long-acting injectable antiretroviral therapies

**DOI:** 10.1371/journal.pone.0351213

**Published:** 2026-06-12

**Authors:** Beth Bourdeau, Alicia T. Bolton, Michelle Palomares, Jonathan Van Nuys, Erin Moore, Gregory M. Rebchook, Mallory O. Johnson, Starley B. Shade, Jesse O’Shea, Kate Buchacz, Kashif Iqbal, Parya Saberi

**Affiliations:** 1 Department of Medicine, University of California, San Francisco, California, United States of America; 2 Division of HIV Prevention, Centers for Disease Control and Prevention, Atlanta, Georgia, United States of America; Pan American Health Organization, UNITED STATES OF AMERICA

## Abstract

**Background:**

Clinic-based administration of long-acting injectable antiretroviral therapy (LAI-ART) is resource-intensive and may exacerbate disparities in access to care. Home-based administration by trained layperson injectors, or treatment buddies (TBYs), could expand access to LAI-ART; however, maintaining high-quality clinical care requires selecting suitable TBYs for its implementation. This paper describes the development of the TBY Selection Tool to support people with HIV (PWH) in identifying appropriate TBYs.

**Methods:**

The tool development process consisted of 5 phases. In Phase 1, semi-structured interviews were conducted with 19 clinicians, 16 PWH, and 15 candidate TBYs. Transcripts were thematically coded to identify domains influencing TBY suitability. In Phase 2, draft items were developed and refined into a structured survey. In Phase 3, 7 PWH completed the tool during a 2-month pilot. In Phase 4, a Community Advisory Panel (CAP) (n = 10) reviewed items for clarity, relevance, and comprehensiveness. In Phase 5, feedback was incorporated to finalize the tool.

**Results:**

Clinicians emphasized the reliability of TBYs, the confidentiality of home-based injections, and the importance of adhering to injection schedules. PWH prioritized trust in the TBYs, TBY availability, and comfort with bodily intimacy. TBYs highlighted emotional steadiness, willingness to learn, and responsibility for continuity of care. These domains were operationalized into a survey assessing relationship history, reliability, confidentiality, availability, proximity, and comfort with injections. Pilot testing showed 100% completion without difficulties. CAP feedback led to refined wording, expanded response options, and clearer phrasing, which were used to finalize a 12-item survey.

**Conclusions:**

The TBY Selection Tool provides a structured framework to support PWH in identifying appropriate TBYs for home-based LAI-ART. By integrating clinical, individual, and community perspectives, the tool addresses factors important for the safe, acceptable, and feasible implementation of home-based LAI-ART. Psychometric assessment, scoring, and further validation in larger, more diverse populations is needed.

## Introduction

Approximately 60% of people with HIV (PWH) in the United States achieve viral suppression [1,2], leaving a significant gap in treatment outcomes due to barriers such as pill fatigue, dosing requirements, stigma, and inequitable care delivery [3]. Long-acting injectable antiretroviral therapy (LAI-ART), such as cabotegravir/rilpivirine (CAB/RPV) administered every 4 or 8 weeks, provides a promising alternative to daily oral ART by addressing some adherence challenges [4–6]. While studies demonstrate LAI-ART’s effectiveness and acceptability across diverse populations [7–15], its clinic-based delivery remains resource-intensive, requiring frequent visits, dedicated personnel, cold-chain storage, and increased clinic capacity [3,16]. These logistical and structural demands may widen existing healthcare disparities, particularly for individuals facing barriers, such as stigma, financial constraints, and limited access to care [3,6,17,18]. Thus, alternative delivery models that reduce reliance on clinics are critical to ensuring equitable access and maximizing the public health impact of LAI-ART.

The 2022 National HIV/AIDS Strategy prioritized therapies like LAI-ART to address the 35% of PWH who remain unsuppressed [2,19], underscoring the need for innovative approaches to scale up its use. Home-based administration of LAI-ART has emerged as a feasible and acceptable alternative to clinic-based delivery, helping to address specific barriers, such as stigma and logistical challenges. Evidence from other therapeutic areas (contraceptive self-injection [20,21], diabetes [22], asthma [23], epinephrine for the treatment of anaphylaxis [24], and home-based administration of multidrug-resistant tuberculosis therapies [25,26]) demonstrates the safety, cost-effectiveness, and patient satisfaction of decentralized drug delivery models. Similar to successes previously seen with community healthcare workers for home-based HIV care [27], preliminary studies on LAI-ART have also shown promising results. For example, projects from the Medical University of South Carolina and the Whitman-Walker Medical Clinic in Washington, D.C., achieved high rates of viral suppression with a home-based model using healthcare workers [28,29]. Additional studies indicate that in-home administration aligns with patient preferences for privacy, convenience, and autonomy [30–33] while reducing clinic burdens. Additionally, allowing PWH virally suppressed on LAI-ART to transition to home-based administration by trusted partners, family members, or trained laypersons [34,35] could ease clinic burdens and support broader adoption [6,36,37,38]. These findings highlight the potential of home-based delivery to improve adherence and expand access to populations disproportionately affected by HIV and those in rural areas [39], improving equitable access. Home-based delivery can help maximize the public health impact of LAI-ART and improve health outcomes for PWH.

The Innovative Administration of Long-Acting Injectables for HIV Treatment Enhancement at Home (INVITE-Home) study [40] aims to address barriers to HIV treatment by expanding LAI-ART delivery to include home-based administration by trained, non-medical layperson injectors, referred to as “treatment buddies” (TBYs). Formative research gathered insights from HIV clinicians [41], PWH, and TBY candidates [42], guided the development of a training curriculum [43], and addressed implementation factors using the Implementation Research Logic Model (IRLM) [44,45]. The training incorporates principles of adult learning (andragogy) [46] to ensure patient and TBY safety while integrating home-based services into clinical workflows. By grounding the training in the needs and preferences of PWH and their chosen TBYs, INVITE-Home seeks to provide an innovative, patient-centered approach to HIV treatment that enhances accessibility and reduces logistical challenges. Selecting an appropriate and reliable TBY is important to the success of this approach. In this paper, we describe the development of a tool to inform TBY selection.

## Methods

**Fig 1:**
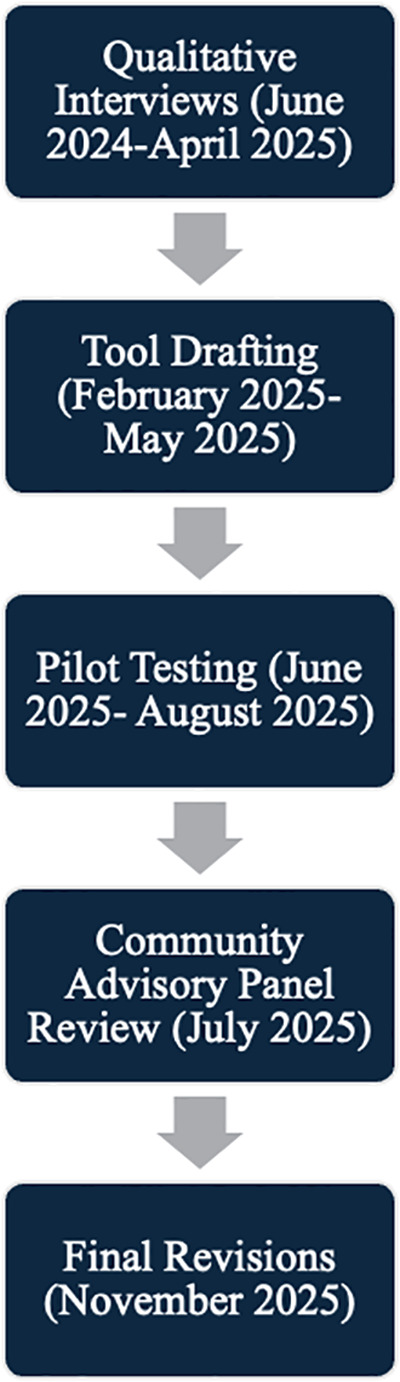
Treatment buddy selection tool development process.

Guidance on choosing a TBY was a critical goal of the formative research, with the objective being a tool for use during recruitment in the full implementation-effectiveness INVITE-Home study. The process of developing the TBY Selection Tool (see Fig 1) began with the steps to draft the initial tool, including: conducting and analyzing qualitative interviews (**Phase 1**); drafting the tool (**Phase 2**); pilot testing (**Phase 3**); review by a Community Advisory Panel (**Phase 4**); and final revisions (**Phase 5**).

### Phase 1: Qualitative interviews

We conducted semi-structured, qualitative, formative interviews with PWH and clinicians from 4 partnering clinics. PWH were asked to select one person whom they would consider asking to provide their injections (i.e., candidate TBY), and those candidate TBYs were also interviewed. All participants were asked to identify preferences and considerations for choosing a candidate TBY for home-based LAI-ART.

Clinic leadership identified clinicians engaged in LAI-ART, who were then purposively sampled across diverse roles, including physicians, pharmacists, nurse practitioners, nurses, and social workers. Interviewed clinicians played key roles in LAI-ART provision, including eligibility assessment, insurance authorization, medication ordering and storage, injection administration, and adherence monitoring. Clinicians were eligible if they worked at a partnering clinic and were closely involved in the clinic’s injectable program or were willing to consider referring patients on LAI-ART to the INVITE-Home study.

PWH were recruited through clinician referral and study flyers. PWH were eligible if they were aged ≥18 years, receiving care at a partnering clinic, had been prescribed injectable CAB/RPV for HIV treatment, were proficient in English, and could identify a TBY to whom they had disclosed their HIV status and who would be willing to consider administering injections. Eligible PWH provided contact information for a candidate TBY, who was then contacted by study staff. TBYs were eligible if they were aged ≥18 years, were proficient in English, identified by a participating PWH, and willing to consider providing LAI-ART injections.

All participants provided verbal consent. Interviews were conducted before any home-injection activities. The study was approved by the University of California, San Francisco IRB (IRB #23–40016). Semi-structured, one-on-one interviews were conducted virtually via a Health Insurance Portability and Accountability Act (HIPAA)–compliant version of Zoom between June 2024 and April 2025 by a qualitative researcher and the study clinician. Interviews lasted 45–60 minutes, were audio recorded, and participants were offered a $40 e-gift card. Field notes were taken to capture participant perspectives and contextual details not evident in the audio recordings or transcripts. Audio recordings were transcribed using Zoom’s automated transcription feature, reviewed for accuracy, and de-identified by study staff. Each participant was assigned a unique identifier.

Semi-structured qualitative interview guides were developed by the study team and informed by the study aims. Separate guides were developed for clinicians, PWH, and TBYs, with overlapping domains across groups. Interview topics included experiences with LAI-ART, acceptability of home-based administration, anticipated barriers and facilitators, training needs and preferences, learning environment and support, and considerations related to safety, monitoring, and communication between the study team, the clinic, and the PWH.

Although PWH and TBY participants were recruited as pairs, interviews were conducted and analyzed separately by participant role to capture distinct perspectives and inform role-specific insights. Dyadic relationships were not systematically analyzed, as this formative work aimed to center individual experiences and avoid over-interpretation of interpersonal dynamics. Interviews were analyzed separately for each participant group (clinicians, PWH, and TBYs) using a combination of inductive and deductive thematic analysis to capture the distinct roles, responsibilities, and decision-making contexts within each group. Examining each group independently allowed themes unique to their experiences to emerge, while also preventing perspectives from being collapsed or conflated across fundamentally different positions within the intervention. De-identified transcripts were thematically coded in Dedoose [47], a web-based, qualitative data analysis platform, by the qualitative researcher using a codebook developed from transcript review and refined iteratively with the study team. Transcripts were secondarily coded using Versa, the University of California, San Francisco’s secure, generative artificial intelligence (AI) platform. Manual and AI-assisted coding were compared in structured tables, and discrepancies reconciled. Themes were finalized once saturation was reached, with illustrative quotes selected to represent constructs that directly informed the development of the TBY Selection Tool.

### Phase 2: Drafting the tool

Using the qualitative interviews, the study team independently drafted initial lists of key domains, based on their field notes and observations. During a reconciliation meeting, the two drafts were side-by-side mapped to identify overlaps, discuss divergent emphases, and determine which items warranted inclusion. Decisions were guided by the prevalence and salience of themes across interviews and their relevance to supporting PWH in selecting a TBY.

The reconciled version was then used to develop a structured survey with categorical and scaled responses across the domains. Subsequent reviews streamlined wording, harmonized scales, and reduced redundancy to keep the instrument concise and analytically sound.

The quantitative researchers reviewed and edited the tool through a group consensus process involving a team with expertise in quantitative and qualitative research, clinical care, and psychology. The team again reviewed the tool to reduce redundancies, enable delivery via written surveys, improve question comprehension, and incorporate best practices for Likert scale response options [48].

### Phase 3: Pilot testing

The study team enrolled participants in a 2-month pilot of the implementation study [40] from June through September 2025. Pilot study participants were referred from partnering clinics, some of whom had engaged in qualitative interviews in Phase 1. PWH were eligible if they were aged ≥18 years, receiving care at a partnering clinic, had been prescribed injectable CAB/RPV for HIV treatment, were proficient in English, and could identify a TBY to whom they had disclosed their HIV status and who would be willing to administer injections. At the enrollment appointment, PWH provided informed consent, completed the TBY Selection tool, and then provided contact information for a candidate TBY, who was contacted by study staff for recruitment into the intervention. All data collection was conducted via a confidential REDCap survey link. The pilot study was approved by the University of California, San Francisco IRB (IRB #24–42341).

### Phase 4: Community advisory panel

All participants in the Phase 1 qualitative interviews were invited to join the INVITE-Home Community Advisory Panel (CAP). The 2-hour CAP meeting took place on Zoom in July 2025. The study coordinator sent a weblink to a test TBY Selection Tool REDCap survey to CAP members before the meeting. During the meeting, the facilitator presented each question in order, soliciting input from CAP members on both the question and the response options, including ease of understanding, comfort with answering, and adequacy of the response options. They were also asked about the order of the questions, redundancy, and whether any domains were not addressed in the current draft. The meeting was audio recorded, and participants were offered a $125 e-gift card.

### Phase 5: Final revisions

Following the CAP meeting, the study team reviewed the notes and audio recordings to incorporate all feedback into a final round of edits.

## Results

### Phase 1: Qualitative interviews

#### Participants.

Nineteen clinicians participated (7 physicians, 4 pharmacists, 4 registered nurses, 2 nurse practitioners, and 2 social workers). Thirty-one interviews were conducted with PWH and candidate TBYs (16 PWH, 15 TBYs), resulting in 15 complete duos and 1 individual PWH whose TBY could not be reached (see Table 1 for PWH/TBY participant characteristics). Key quotes from clinicians, PWH, and TBYs are included in Table 2; exemplar quotes are noted by table row number.

**Table 1:** Demographic characteristics of participants in Phases 1, 3, and 4.

	Phase 1: Qualitative Interviews (n = 50)	Phase 3: Pilot Testing (n = 7)	Phase 4: Community Advisory Panel (n = 10)
**Characteristic**	**n (%)** ^**†**^	**n (%)**	**n (%)**
Participant type			
Clinician	19 (38)*	N/A	3 (30)
PWH/TBY	31 (62) ^**†**^	7 (100)	7 (70)
Race/ethnicity^‡^			
Black/African American	10 (32) ^**†**^	1 (14)	3 (30)
White	10 (32) ^**†**^	5 (71)	3 (30)
Asian	2 (6) ^**†**^	1 (14)	2 (20)
Middle Eastern/North African	- ^**†**^	–	1 (10)
Hispanic/Latino	4 (13) ^**†**^	–	1 (10)
Multiracial	2 (6) ^**†**^	–	–
Missing	3 (10) ^**†**^	–	–
Sex			
Male	17 (55) ^**†**^	5 (71)	4 (40)
Female	11 (35) ^**†**^	2 (29)	6 (60)
Missing	3 (10) ^**†**^	–	–
Sexual orientation			
Gay or lesbian	12 (39) ^**†**^	4 (57)	3 (30)
Heterosexual or straight	15 (48) ^**†**^	2 (29)	6 (60)
Bisexual/pansexual	1 (3) ^**†**^	1 (14)	1 (10)
Missing	3 (10) ^**†**^	–	–
Highest level of school completed			
Less than a high school diploma	5 (16) ^**†**^	2 (29)	–
High school graduate/GED	5 (16) ^**†**^	–	1 (10)
Some college	9 (29) ^**†**^	4 (57)	3 (30)
College graduate or above	9 (29) ^**†**^	1 (14)	6 (60)
Missing	3 (10) ^**†**^	–	–
Median age (range)	53.5 (33-74) ^**†**^	54 (46-64)	49 (39-61)
Relationship to TBY	N/A		N/A
Significant other/spouse		3 (43)	
Family member		1 (14)	
Friend		2 (29)	
Neighbor		1 (14)	
Other		–	
Median relationship years (range)		6 (1.5-35)	

* Demographic data not provided by clinicians.

† Percentages provided for PWH/TBY only.

‡ Participants allowed to choose more than one option.

GED: General Educational Development; N/A: not applicable; PWH: people with HIV; TBY: treatment buddy.

**Table 2:** Illustrative quotes highlighting participant perspectives on training needs for home-based LAI-ART.

Participant group		Participant (ID & demographics)	Illustrative quote
Clinician quotes	1.	Physician	“A lot of my patients are really socially isolated, and I just don’t think they have this kind of person in their life.”
	2.	Physician	“a stable presence … somebody who is able to be responsive, has a phone, [and] some degree of organizational skills …. The pattern of past stability is something to take into consideration.”
	3.	Physician	“If you have a really hard time reaching them over the phone and they don’t return calls or messages... that’s a fair indicator the strategy could not work that well.”
	4.	Physician	“They have to be someone they’ve disclosed their status to who can be confidential …. It’s definitely a major concern of some of our patients.”
	5.	Nurse Practitioner	“Does the treatment buddy show up when they’re supposed to? Is this relationship sustainable? Is it a transitory relationship?”
	6.	Registered Nurse	“Is the person really, truly reliable to be there with you every two months or every month to inject the medication?”
	7.	Pharmacist	“Someone who first of all is interested and invested in learning the technique correctly and learning all the nuts and bolts of [medication] storage and drawing it up, someone who’s reliable … [and] willingness is really what it takes …. Reliability, willingness to learn, sense of commitment, and everything else will fall into place.”
	8.	Registered Nurse	“Does that person have any medical issues that might keep them from giving the injection? Do they have any problems with dexterity, or do they have shakes?”
	9.	Social Worker	“…someone that is in remission, not using.”
People with HIV (PWH) quotes	10.	61-year-old, White, male	“…someone who genuinely cares about you, has a commitment to the relationship that you have, whatever the nature of that relationship might be … a certain degree of stability … and not a turbulent relationship.”
	11.	53-year-old, White male	“I trust she’d be quiet, cause my mother-in-law doesn’t know that I have HIV. I trust my sister-in-law greatly to keep it between us.”
	12.	55-year-old, White, female	“Definitely somebody that’s very close to me and knows my status. I’m pretty open with it, but just knowing how important it is, that’s huge …. It’s a must. It’s super important.”
	13.	65-year-old, Hispanic/Latino, male	“My son, he’s a great kid, but … he never answers his phone …. My daughter, she’ll answer the phone. She’ll get back to you right away. She won’t wait two hours …. She has my back.... She’s always on point, on time …. Anytime you need her, she’s there.”
	14.	58-year-old, Black/African American, female	“I was gonna have try to do this with my ex, he don’t wake up till late, and he’s hard to get a hold of. I need somebody I can rely on when it comes to this.... He’ll never answer his phone, and you have to be on top of this. This is important. He’s not reliable, and I need somebody reliable.”
	15.	53-year-old, White, male	at least six months.”
	16.	61-year-old, White, male	“Someone that they likely will continue to have contact with and be in proximity with for an extended period of time.”
	17.	36-year-old, Black/African American, male	“… you know they’re going to be around for a year.”
	18.	53-year-old, White, male	“Reliability. You have to make sure that they’re able to give the shot the time and day that you need the shot done.”
	19.	65-year-old, Hispanic/Latino, male	“I want to make sure they show up, and they’re on time for it and they ain’t been drinking, or something.”
	20.	61-year-old, White male	“If the buddy is someone who uses drugs … their life condition may be a bit unstable …. I wouldn’t want them to be altered mental status or intoxicated when they’re doing the injection.”
	21.	61-year-old, White male	“Consistently having contact with that person. If that person is someone who lives in another city that the patient may see once a month or once every two months, that would be a bit of a red flag.”
	22.	45-year-old, White, male	“It would be somebody that you’d normally see on a daily or weekly basis …. Somebody that you know that’s around and is dedicated to be there for you.”
	23.	53-year-old, White male	“You have to make sure the person has transportation to your hour and get there on the designated time for the shot.”
	24.	46-year-old, Black/African American, female	“It’d be nice to have your kids do it, but then, at the same time, do you really want your kids to see your behind? So, you gotta think about that …. The trust level got to be there because you’re gonna be vulnerable. You’re gonna have to bend over.”
	25.	53-year-old, White, male	“You’re gonna see someone’s behind …. You’re gonna see some personal stuff that [you] usually won’t show to just anyone.”
	26.	61-year-old, White, male	“Someone who is squeamish about medical things, or needles, or blood.”
	27.	55-year-old, White, female	“You have to be comfortable around needles …. I want somebody to be able to not have to go twice, and like try it again. It’s a one-shot deal on each side.”
	28.	61-year-old, White, male	“Someone who has the mental capacity.”
	29.	59-year-old, White, male	“Emotionally and psychologically stable, because if they’re not … they can really fluctuate …. Are they emotionally able to do it on a given day? Some days, they may be able to. Some days, they may not be able to physically. I think good hand dexterity is going to be good.”
	30.	59-year-old, White, male	“Picking the right person is really important here, ‘cause it can make or break the whole deal.”
Treatment Buddy (TBY) quotes	31.	Age not reported, White, male	“Someone who’s reliable. Like, really reliable that they’re there for you whenever, and they stick to their word. No shenanigans. Someone who understands how important it is to give the injection and to get it and the timing of it …. Basically, someone who’s gonna show up and show up on time and show up in the in the proper state.”
	32.	63-year-old, Black/African American, male	“It comes down to just straight trust, love, and trust, knowing that person to be there for you. Period … I’m gonna be there for my wife, no matter what. Whatever it takes for me to learn to help her.”
	33.	54-year-old, Black/African American, female	“Do you trust them? Do you want them to know your situation? How long you been knowing this person, because everybody don’t want everybody to know they’re HIV positive, so you have to be really careful of who you ask.”
	34.	41-year-old, White, male	“You would want to be comfortable with the person, considering where the site is.”
	35.	38-year-old, Hispanic/Latina, female	“Definitely somebody who’s not scared to do it. Number one, right. Like, seeing how they actually feel about it …. I think the biggest thing is the person not being scared to do it, or fearful of the process.”
	36.	Age not reported, race/ethnicity not reported, female	“I think you should know the person for a while and be comfortable with them. That’s why my friend asked me if I would want to do this, because we’re friends, we’ve known each other for a couple of years.”
	37.	57-year-old, Hispanic/Latina, female	“It has to be somebody who they feel very comfortable with and speaking about their health concerns. I know why (PWH)’s chosen me. Not only are we good friends, but I’ve been through some of his health issues. I’ve been with him, I’ve held his hand, I’ve listened to the doctors, I’ve advocated for him …. It’s somebody who feels comfortable being in that role medically …. I think a big part of it is really feeling comfortable with the person’s medical condition, and that they feel that they can handle all of that comes with it … [and] … [PWH] needs to feel comfortable with me. He has to feel comfortable with me, too.”
	38.	Age not reported, White, male	“at least six months.”
	39.	Age not reported, race/ethnicity not reported, male	“…a year.”
	40.	56-year-old, Multiracial, female	“For sure, they need to make sure they’re comfortable with the patient. They’re not afraid to give injections, other than learning and getting used to it... they can learn for sure. They [need to] understand the technique, making sure it’s safe, understand why they’re giving it, what is it for, [and] how is it benefiting.”
	41.	51-year-old, Black/African American, female	“Someone who they’re comfortable with, who’s nonjudgmental, who’s open-minded, understanding, isn’t afraid of needles, who understands confidentiality, that’s the main thing.”
	42.	53-year-old, Black/African American, male	“I never wanna mess up …. My whole design is just not messing up …. Let’s just say that I started doing injections for my partner and then, for whatever reason, I can’t do it. Do they have to have a backup buddy?”
	43.	Age not reported, race/ethnicity not reported, male	“What would be the concern of having to change your partner, like if your partner is no longer available, and I need a plan?”

**Clinician insights:** Clinicians emphasized several core considerations they believed were essential for identifying a suitable TBY for home-based LAI-ART, focusing on reliability, stability, confidentiality, and the individual’s ability to safely perform injections. Clinicians anticipated that some PWH would struggle to identify a suitable TBY because of isolation and limited networks (#1). They emphasized stability, reachability, and follow-through as essential qualities for this role (#2 and #3). Confidentiality was also central, with clinicians noting that a TBY must be someone to whom the patient has safely disclosed their HIV status (#4).

Reliability and long-term commitment were repeatedly underscored, particularly given the strict timing of injections (#5 and #6). Clinicians also stressed the importance of willingness and skill acquisition, highlighting the need for individuals who are open to learning, confident around needles, and capable of handling medication storage and preparation (#7).

Finally, clinicians cautioned that health-related limitations could compromise safe injection delivery. They noted the importance of screening for issues, such as dexterity, tremors, or other health conditions that might impede injection ability (#8), as well as ensuring the TBY was stable in their own recovery if they had a history of substance use (#9).

**PWH insights:** PWH emphasized that choosing a TBY required balancing relational trust, stability, communication, and practical reliability, alongside considerations of physical intimacy and the TBY’s emotional and physical readiness for the role. PWH considered selecting the right TBY as pivotal to the success of home-based LAI-ART. While practical issues, such as availability and proximity, mattered, participants’ decisions were deeply personal, rooted in trust, stability, and confidence in the TBY’s commitment (#10). These qualities were described as the foundation for all considerations in terms of the TBY’s discretion within the home (#11) and their knowledge of the PWH’s HIV status (#12). Communication and responsiveness were also highlighted as critical. Participants contrasted reliable contacts who responded quickly with others who were inconsistent and hard to reach, noting that timeliness was crucial to maintaining the injection schedule (#13 and #14).

Established relationships provided reassurance, though participants were divided on whether a minimum relationship duration was necessary. Some suggested knowing the person for at least 6 months before considering them as a TBY (#15), while others emphasized that it was not just about the length of the relationship, but confidence in the person’s future presence in their life (#16 and #17).

Reliability was identified as an essential criterion. Participants expressed concern about instability, substance use, or inconsistent communication interfering with the TBY role (#18, #19, and #20). Proximity and regular contact were also important, with participants emphasizing that TBYs needed to live nearby (#21 and #22) and have reliable transportation (#23).

Comfort with physical intimacy, given that injections are administered in the buttocks, was another factor. Participants emphasized the importance of trust and openness, particularly when receiving an intramuscular injection in the gluteal area (#24 and #25). Barriers, such as squeamishness, fear, and incapacity, were seen as making someone unsuitable for the role (#26 and #27). Emotional steadiness and physical dexterity were also essential (#28 and #29). Ultimately, selecting a TBY was far from a casual decision. It required honest reflection on emotional readiness, long-term stability, and mutual respect. Several highlighted it as a high-stakes choice that could determine whether home-based injections succeeded or faltered, underscoring that the “right” person was essential to making the entire model work (#30).

**TBY insights:** TBYs highlighted dependability, trust, confidentiality, comfort with the injection process, emotional steadiness, relationship history, and a clear understanding of their responsibilities as key considerations that shaped their readiness to serve in this role. Dependability and punctuality were described as central, requiring not only showing up but doing so consistently, on time, and reliably (#31). Trust, often grounded in love and commitment, was also described as fundamental (#32). Confidentiality was a critical component of trust (#33).

Comfort with the injection process and privacy, given the location of the injections on the body, were considered essential (#34). TBYs emphasized the importance of being emotionally steady, confident, and unafraid of the technical or personal aspects of administering an injection, particularly avoiding fear or hesitation (#35).

Relationship history and continuity provided additional reassurance. Established relationships, familiarity over time, and shared health experiences strengthened TBY’s assessment of their ability to serve in this role (#36 and #37). Like PWH, TBYs also suggested that a minimum relationship length was important before taking on the role (#38 and #39). Beyond their relationship history, TBYs stressed the importance of understanding safe injection technique and the treatment’s purpose and significance (#40).

Finally, TBYs underscored the importance of emotional steadiness, open-mindedness, and a non-judgmental stance as essential qualities in a TBY (#41). They also reflected on the weight of responsibility the role carries, emphasizing the pressure to administer injections correctly and the need for contingency planning (#42 and #43). These reflections highlighted that, beyond emotional readiness, backup arrangements were viewed as essential to ensuring continuity of care.

### Phase 2: Drafting the tool

Results from the reconciled list of key domains included: trust and confidentiality; reliability and availability; commitment to the injection schedule over the planned 12-month study period; timeliness and responsibility; reachability and stability; training and capability; physical proximity and comfort during injections; and transparency and communication. Each key domain was first used to develop open-ended questions, after which team members with quantitative and survey development expertise were brought in to convert each into a closed-ended question with Likert responses. The list of items was reviewed multiple times to improve language precision, clarify differences among similar items, and reduce redundancy. Team experts felt that some items required concrete examples (e.g., providing a ride to the airport or caring for a pet) to convey the level of gravity or importance for assessments of the TBY’s reliability or availability.

We refined response categories so that most used a unipolar, five-point Likert scale, ranging from 1 (low) to 5 (high) (e.g., “1 - Not at all” to “5 - Extremely”) [48,49]. Two questions about the TBY’s geographical proximity to the PWH and their response time when contacted did not use Likert scales. “Proximity” was measured in terms of typical travel time rather than mileage, and an “ease-of-contact” item was added to capture how quickly a TBY usually replies to any form of communication.

This process led to the identification of several improvements. Eight of the initial 17 questions were removed, and 4 were modified. Four questions were added about the length and type of relationship with the TBY, the TBY’s comfort with seeing blood, and the person’s trust that the TBY will keep health information confidential (see Table 3 for the final version of the TBY Selection Tool).

**Table 3:** Finalized treatment buddy selection tool.

Questions	Response options
1. What is your relationship with the person you are considering for your treatment buddy? Select all that apply.	1. Significant other (e.g., partner, spouse, boyfriend, girlfriend) 2. Family member (e.g., parent, child, sibling, aunt/uncle, cousin, etc.) 3. Friend 4. Roommate 5. Neighbor 6. Caregiver 7. Other 8. Prefer not to respond
2. How long have you known this person?	1. Less than 1 year a. How many months have you known them? ____ 2. One or more years a. Approximately how many years have you known them? ____ 3. Don’t know 4. Prefer not to respond
3. How comfortable are you discussing your HIV treatment with this individual?	1. Extremely comfortable 2. Very comfortable 3. Moderately comfortable 4. Slightly comfortable 5. Not comfortable 6. Not sure 7. Prefer not to respond
4. How much do you trust this person to keep your medical information confidential?	1. Completely 2. Very much 3. Moderately 4. A little 5. Not at all 6. Not sure 7. Prefer not to respond
5. How willing are you to let this individual see and touch the area of your buttocks where you get your injections?	1. Extremely willing 2. Very willing 3. Moderately willing 4. Slightly willing 5. Not willing 6. Not sure 7. Prefer not to respond
6. How much can you rely on this individual to consistently meet commitments? E.g., if you needed someone to drive you to the airport or an appointment or take care of your pet, do you think you could rely on this person?	1. A great deal 2. A lot 3. A moderate amount 4. A little 5. Not at all 6. Don’t know 7. Prefer not to respond
7. How well does this person’s schedule (e.g., work, school, volunteer commitments, or any travel or moving plans) fit with your injection schedule over the next 12 months? Think about whether the person will be able to show up consistently for your monthly or bimonthly injections.	1. Definitely fits 2. Probably fits 3. Might or might not fit 4. Probably won’t fit 5. Definitely won’t fit 6. Not sure 7. Prefer not to respond
8. If you need to reach this person about something important (e.g., by text or phone call), how quickly do they typically respond?	1. Less than one hour 2. Within a few hours 3. Within a day 4. Within two days 5. Longer than two days 6. Not sure 7. Prefer not to respond
9. How long does it typically take for this person to get to you from where they live? Consider their travel time using their typical mode of transportation, such as driving or taking a bus.	1. They live in the same residence (no travel time needed) 2. < 10 minutes 3. 10–20 minutes 4. 20–30 minutes 5. 30–60 minutes 6. > 60 minutes 7. Not sure 8. Prefer not to respond
10. How comfortable do you think this individual is with needles and administering injections? E.g., anxiety or phobia around needles or triggers from previous injection experience	1. Extremely comfortable 2. Very comfortable 3. Moderately comfortable 4. Slightly comfortable 5. Not comfortable 6. Don’t know 7. Prefer not to respond
11. How comfortable do you think this individual would be with seeing a few drops of blood at the injection site?	1. Extremely comfortable 2. Very comfortable 3. Moderately comfortable 4. Slightly comfortable 5. Not comfortable 6. Don’t know 7. Prefer not to respond
12. How often does this person have a physical limitation that might impact their ability to give injections? E.g., tremors, nerve/muscular disorders, arthritis, or other grip strength challenges	1. Always 2. Very Often 3. Sometimes 4. Rarely 5. Never 6. Don’t know 7. Prefer not to respond

### Phase 3: Pilot testing

Seven PWH completed the TBY Selection Tool during the pilot period (see Table 1 for participant information). The completion rate was 100%, and no participant reported difficulties with the instrument. Participants were not provided with any summary information regarding their responses to the tool, but study staff asked whether they were reconsidering their chosen TBY after completing the survey.

Given the small sample size, no additional calculations or conversions were made to the data, and no items needed reverse scoring prior to analysis. No summative overall or threshold scores were assessed, leaving those analyses to the planned implementation-effectiveness study. Simple descriptive statistics were used to assess data quality. All PWH had known their TBY for over a year, with 3 TBYs described as a significant other, 2 as a friend, 1 as a family member, and 1 as a neighbor. PWH assigned their selected TBYs high scores for nearly all questions, reflecting the high certainty of people’s choice during the pilot period. No participant responded with a score lower than 3 (on a scale from 1 to 5, with 1 being the lowest). Ceiling effects [50] will be assessed in the larger study, and data transformations will be applied as indicated.

### Phase 4: Community advisory panel

Ten people participated in the CAP (Table 1), including 3 clinicians and 7 non-clinicians (PWH or TBYs). Participants recommended expanding relationship options to include “adult child,” and the trust item to be narrowed from general care to HIV care and injections. Participants requested edits to the wording of response scales (e.g., “Not comfortable” rather than “Not at all comfortable”) to improve comprehension. They expressed that the language around bodily intimacy during injections should be reframed from “comfort” to “willingness” to capture readiness despite potential discomfort better. Additionally, the anatomical site was clarified as the “injection area of the buttocks.” They had additional suggestions for the examples used to assess a TBY’s reliability (e.g., “drive you to an appointment”) as well as for the capability item (e.g., physical limitations such as arthritis, decreased grip strength, weakness). They recommended a companion item related to capability for non-physical considerations (e.g., anxiety, needle phobia, triggers). “A small amount of blood” was clarified as “a few drops.” They also felt that the “ease-of-contact” item needed additional specificity for both text and phone, with an additional response option (<1 hour). They requested that proximity be reframed in terms of travel time, accounting for typical modes of transportation.

### Phase 5: Final revisions

Final edits included removing 1 question for redundancy and rearranging the questions in the order of importance. With these refinements, the resulting 12-item instrument became brief, concrete, and easy to administer (the final questions and response options are available in Table 3). It integrated clinical priorities and community guidance, offering indicators of TBY suitability for home-based LAI-ART and clear touchpoints for discussion when potential concerns arose.

## Discussion

The TBY Selection Tool translates patient and clinician priorities into a practical instrument that can support safe home-based LAI-ART and broaden access to person-centered care. Drawing on proven models from other areas of healthcare, such as hormone therapy and fertility care, home-based administration by using TBYs offers a decentralized approach to delivering LAI-ART, helping to overcome barriers to treatment uptake and long-term adherence. This initiative seeks to broaden access to flexible, person-centered care while enhancing the practical effectiveness of LAI-ART. It aims to empower PWH with greater convenience, privacy, and autonomy in managing their care by using trained TBYs and combining behavioral and biomedical strategies. Through the formative work, it was clear that selecting the TBY would be essential to the success of this approach. High confidence in PWH’s choice of their TBY is necessary for the future success of a home-based LAI-ART program.

Across participant groups, there was strong alignment in identifying trust, confidentiality, reliability, and consistency as core characteristics of a suitable TBY. However, perspectives differed in emphasis. Clinicians more frequently focused on clinical safety, physical capability, and adherence to injection schedules, while PWH and TBYs emphasized relational trust, communication, emotional readiness, and the realities of maintaining these roles within everyday life. PWH highlighted the deeply personal and high-stakes nature of selecting a TBY, while TBYs underscored the responsibility and pressure associated with the role. These differences point to the need for approaches that address both clinical and interpersonal dimensions of TBY selection. Transition to home-based administration would likely involve shared decision-making, and it is imperative that both the patient and the clinician feel confident that the patient’s care will be maintained. The full study, with results indicating potential clinical thresholds, could be an important tool in that decision-making process.

The development of the TBY Selection Tool paralleled other work within and outside of the field of HIV treatment research. Notably, there is a rich literature on romantic relationships and support (from formal caregiving to informal support) where relationship stability, trust, and confidentiality are central to treatment success across a range of health and illness contexts [51]. The nuances of social support and its effects on outcomes have been the subject of health-related research for decades. For example, research has shown that the presence of problematic support can result in greater depressive symptoms among patients with rheumatoid arthritis, whereas positive support is predictive of reduced symptoms of distress [52]. The degree of training and preparation provided to caregivers has also been shown to be predictive of outcomes, including perceived burden and burnout among caregivers in the context of stroke rehabilitation [53]. Within the context of HIV treatment adherence among couples, a partner’s characteristics, such as lower depressive symptoms, greater relationship satisfaction, and stronger beliefs about treatment necessity, have been linked to better treatment adherence and virologic control [54]. Relatedly, previous research has identified important characteristics of an effective peer in emergency departments [55] and HIV clinical settings [56]. These include intrinsic qualities (e.g., reliable, adaptable, a good listener), shared experiences, personal stability, proximity, and disclosure of HIV status.

While there are differences in the expected roles of a romantic partner and a proposed TBY in the context of treatment injections, several elements emerged in the formative work that overlap with the broader literature on relationships and health. Within the context of HIV treatment and couples research, other studies have identified similar qualities of romantic relationships, including relationship satisfaction, trust, and intimacy, that have been linked to treatment [54,57,58]. Future research exploring variations in preferences and outcomes across different types of TBY (e.g., friend, romantic partner, family of origin, or choice) may provide insights into effective selection and training of TBY for patients from different populations, cultures, and backgrounds.

The planned implementation-effectiveness study will test the utility of the TBY Selection tool by assessing its ability to reliably identify TBYs who are more likely to be retained and to maintain high-quality HIV care. We will examine the tool’s utility across several measures of TBY success: process measures such as rates of missed or rescheduled appointments and the number of times an alternate TBY must be selected, as well as PWH medical outcomes, including maintaining viral suppression. Ideally, we will be able to identify a critical threshold in the TBY Selection Tool that ensures the TBY chosen will be available long-term, rescheduling only a few and missing no appointments, and safeguarding viral suppression. This can provide a critical bridge to transition and maintain high-quality medical care, reassuring both patients and their care providers.

### Limitations

Despite the potential of the TBY Selection Tool, several limitations must be acknowledged. The tool was developed as part of the formative work and pilot testing of the INVITE-Home study and has been tested with only 7 participants within a limited setting and timeframe. Consequently, we have not been able to validate the tool’s questions or responses. The low sample size also contributed to low variability in the selection tool results (with no responses below 3) and participant outcomes, preventing analysis of the tool’s internal consistency or validity. Further research is needed to validate the tool across larger, more diverse populations and to evaluate its impact on clinical outcomes, patient satisfaction, and adherence. During the full study, we will continue to use the tool with a larger sample over a 12-month follow-up period in a broader geographic area. This next phase will be critical in refining the TBY Selection Tool and ensuring its applicability in broader contexts to improve access to home-based LAI-ART programs. These limitations highlight opportunities for future research to refine and scale this approach. Ultimately, the TBY Selection Tool offers a potentially practical and scalable solution to address the logistical challenges of home-based LAI-ART delivery. As the HIV treatment landscape evolves, the TBY Selection Tool and other similar tools will be indispensable for ensuring access to innovative therapies, improving treatment outcomes, and empowering PWH to take greater control of their care.

## Conclusions

The development of the TBY Selection Tool represents a critical step toward expanding access to home-based administration of LAI-ART. By systematically addressing the qualities that make a successful TBY—such as trust, reliability, emotional readiness, and logistical suitability—the tool provides a structured framework for identifying individuals capable of supporting home-based care while maintaining the high standards of HIV treatment delivery.

As PWH face barriers such as stigma, clinic burden, and logistical challenges, decentralized models of care, including home-based LAI-ART, offer an innovative and patient-centered solution; however, the success of this approach hinges on selecting a TBY, whose role in injection administration is both practical and deeply personal. Our formative research underscores the importance of tailoring this tool to reflect real-world considerations, including relationship dynamics, physical and emotional preparedness, and confidentiality.

The TBY Selection Tool may facilitate the safe and consistent administration of LAI-ART, supporting clinicians’ confidence in the quality of care outside traditional clinical settings. This confidence is essential for broader adoption of home-based delivery models, particularly in settings with limited healthcare resources or among populations disproportionately affected by barriers to clinic-based care. By empowering PWH with greater autonomy, privacy, and flexibility in their treatment, the tool aligns with the national HIV goals to improve viral suppression rates and enhance HIV care for all.
