# Supplementary material for: “Picking the right person … can make or break the whole deal”: Development of a layperson injector selection tool for administration of home-based long-acting injectable antiretroviral therapies
Source: PLoS One. 2026 Jun 12;21(6):e0351213. doi: 10.1371/journal.pone.0351213 (PMC13262845; doi:10.1371/journal.pone.0351213)
Supplement: Attachment — Submitted filename: Response to Reviewers.docx [file pone.0351213.s002.docx]

Response to Reviewers

**Reviewer #1:** Overall thoughts: This is a well-conducted, methodologically sound study addressing an important gap in LAI-ART implementation. The multi-phase, stakeholder-engaged tool development process is exemplary, and findings have direct practical implications for expanding home-based HIV care. With making minor revisions detailed here, this manuscript will make a strong contribution to PLOS ONE.

*We thank the Reviewer for their helpful comments.*

**Major issues:**

1. Authors should be more explicit in their discussion of limitations of the study and comment on the small sample size of this pilot (n=7) in addition to the fact that all participants are from urban settings (San Francisco Bay Area clinics), as this may affect real world performance

*We have added further details in the discussion section about the limitations of the study, specifically a paragraph at the end of the Discussion section (lines 347 to 368).*

2. Furthermore, authors are encouraged to include preliminary internal consistency measures from the pilot data and outline validation plans for future work to address the lack of reliability analysis from the study.

*This is an important point that we have not yet been able to test with our limited dataset. We have added additional details in the discussion about this limitation and plans for future exploration of these points (see paragraph at the end of the discussion section).*

3. Even though the study generates important data on the subject, information on contextualizing results is missing. Authors should include a section on interpretation of scores and clinical application of results i.e., what exactly does ‘suitable’ or ‘unsuitable’ TBY denote? How should clinicians apply the scores as part of counselling?

*We have added sentences at the end of the first two discussion paragraphs on the applications of this tool, such as those mentioned, that we hope to determine through the larger study.*

Minor issues:

4. Authors state that semi-structured interviews were conducted between June 2024 and April 2025 (lines 103-105), and pilot testing (phase 3, line 136-137) from June to September 2025? Kindly align timeline

*The timelines reflect different phases of the study. The qualitative interviews (Phase 1) were conducted between June 2024 and April 2025, and findings from these interviews informed the development of the tool. Pilot testing (Phase 3) was conducted afterward, from June to September 2025. Figure 1 has been modified to include the dates for each phase, some of which overlap.*

5. Kindly clarify the sampling strategy employed in phase 4 (Community Advisory Panel) and report participation rate

*We have updated the Phase 4 description (first sentence) to note that all Phase 3 participants were invited; we used a convenience sample.*

6. Further discussion is required for all scores greater or equal to 3 under the ceiling effects under the results section

*We have clarified this limitation in the discussion section (the last paragraph before the Conclusions).*

7. Phase 2: Drafting the Tool (results, lines 232-249) could be clearer if presented in chronological order, consider revising.

*The Phase 2 description has been edited to convey the order in which modifications were made in the tool’s initial drafts, throughout the entire section.*

8. Kindly specify the ‘missing’ on Table 1, is it clinicians or TBY?

*We had noted by the (****^†^****) symbol that demographics were collected only from the PWH/TBY participants. We have added the (****^†^****) symbol to each of the demographic categories to denote that the numbers and percentages provided pertain only to PWH/TBY and not to clinicians.*

9. Although the discussion is well-written, it can be further enhanced by addressing cultural considerations in TBY selection and strengthening parallels to dyadic HIV/health literature with specific examples

*We have added more context to the discussion as suggested, with greater anchoring of our findings to the dyadic health literature (with examples).*

10. Lines 40-51 and 53-63 appear repetitive, consider consolidating.

*Those two paragraphs have been consolidated.*

**Reviewer #2:** Thank you for the opportunity to review this manuscript on the development of a treatment buddy (TBY) selection tool for patients receiving long-acting injectable antiretroviral therapy (LAI-ART). This manuscript addresses access, confidentiality and comfort around treatment options for PLWHA, with the goal of having more PLWHA with undetectable status'. While this manuscript adds to the literature about LAI-ART and alternative delivery models, there are several areas of concern which I detail below:

*Thank you for this review and suggestions for further improvement.*

**Major:**

Introduction --

1. lines 53-63 --- seems repetitive and doesn't add much, therefore could be woven into paragraphs 1-2 (lines 29-52). It would be better served to outline any prior buddy selection tools that have been used in this population or others. If this is a novel idea, there should be more background as to why the authors thought this would work in this population.

*We have consolidated the two paragraphs into one.*

Methods --

2. lines 102-110 -- How were the guides developed? Could you provide example questions in a table in the supplement or if you feel important, into the main paper? Were there separate guides for clinicians, patients and TBYs? What topics were asked about (I see briefly on lines 88-89, but that is too brief and should be probably moved to around lines 102)?

*We have added a paragraph describing the topics and questions on the interview guides for each of the three categories of participants (paragraph 4 of this section).*

3. lines 96-101 -- Seems like most patients were paired with their TBYs and you mention dyads later, but no mention of dyadic analysis in your methodology. If you did do dyadic analysis, you should explain more in the Methods. If you did not, I would probably drop mentions of dyads (lines 164 and 289).

*While participants were recruited as PWH-TBY pairs, we did not conduct a formal dyadic analysis. As noted in the Methods, interviews were analyzed separately by participant group (clinicians, PWH, and TBYs) to capture distinct perspectives. We have added clarifying language to make this explicit (starting on line 119) and revised references to “dyads” to “duos” to better reflect the study design.*

4. line 127 -- there needs to be a sentence or two making it clear that the tool is meant to be used by PLHWA to identify TBY because in the pilot test, TBYs were also consented for some reason and it was a little confusing.

*The order of activities was changed so that the PWH completed the TBY Selection Tool before contacting the TBY for participation (end of first paragraph).*

5. lines 141-143 -- the consenting of TBYs for the pilot test is confusing. Perhaps they were consented for another process that is not mentioned in the manuscript. For that reason, either clarify why that needed to happen (what role TBYs played in the pilot test -- did the PLWHA verbally ask the TBYs questions from the selection tool?) or remove.

*The pilot study collected data from both members of the duos; however, no data collected from the TBY is presented in this paper. Mention of consent and data collection from the TBY has been removed for clarity.*

6. lines 148-149 -- More needs to be described about the invitation of Phase 1 participants to the CAP and why that decision was made (recruitment difficulties?). It seems like the CAP served as a group cognitive interview to refine the tool more that an advisory panel. An advisory panel seems like an opportunity to get other stakeholders involved (experts, patient advocacy groups, etc.). Methodologically, the CAP should probably be described more as a cognitive interview vs. panel.

*While this session with the CAP was used to gather members’ input on the TBY selection tool, we have and continue to involve the CAP in various aspects of the research. We had few difficulties in recruiting CAP members because they were clinicians from partnering clinics and PWH/TBYs who were interested in this topic.*

Results --

7. Line 249 -- No mention of how the tool is scored after revision. You mention Likert scales, but scoring and decisions about any reverse coding, etc. need to be described. And how does the scoring help the PLWHA decide to select that person as a TBY or not? That is really important to include.

*This is the intended function of the TBY Selection Tool and will be the analytical goal of the full study. Additional details on how we addressed the small pilot dataset are provided (see second paragraph of Phase 3 [Results]).*

8. Lines 272-277 -- Another opportunity to discuss scoring here. And how do you envision tool use in real-world practice (i.e., does the clinic provide the tool to PLWHA, do they find it online themselves)?

*Please see comment above.*

Discussion --

9. Lines 279-300 -- There needs to be expansion on the similarities and differences between interview findings from clinicians and patients and TBYs, any gaps, what might the study find on expanded testing of the selection tool and how might they envision the rollout of this delivery model?

*We have expanded the Discussion section (second paragraph) to more explicitly synthesize key similarities and differences across clinician, PWH, and TBY perspectives, including areas of alignment and divergence.*

**Minor:**

Introduction --

10. line 34 -- there is no mention of side effects (or lack of) of LAI-ART along with effectiveness and acceptability.

*While we concur that side effects play a role in uptake of injectable ART, our focus here is limited to implementation issues for those interested in and tolerant of injectable ART. A shift in the delivery model will not remedy side effects that are sufficiently problematic as to impact their use.*

11. line 35 -- you might provide a better term than specialized personnel, as it is not clear what is meant (do you mean prescribers?) It begs the question, if specialized personnel are needed in the clinic, how can the treatment be delivered by TBYs?

*This term has been correctly flagged as inaccurate. We have changed it to ‘dedicated’ which better reflects the impact of injectable programs on workflow and staffing.*

12. lines 44-45 --- decentralized drug delivery models as opposed to injection models

*We concur and have made the edit.*

Methods --

13. lines 84-86 -- is it important to name the sites? May de-identify since there are small participant numbers.

*We have removed the clinic names.*

14. line 111 -- thematically analyzed

*We have clarified the analytic approach by specifying that a combination of inductive and deductive analysis was used.*

15. line 116 -- consider adding codebook with themes, subthemes and descriptions to a table in main text or supplement

*The key themes are listed in the first sentence for each participant group; the analytic approach is described in the Methods for Phase 1 in paragraph two.*

16. line 119 -- consider adding saturation table by theme/subtheme into supplement

*We clarified that themes were developed and refined iteratively until saturation was reached.*

Results --

17. line 233 -- study team or coding team members as opposed to analysts

*We have made this edit.*

18. line 234 -- Is "1-year" commitment important? This is the only time it is mentioned and is very specific. Why not just commitment to injection schedule with no time frame?

*We have clarified this requirement as it is the length of the implementation-effectiveness study for which this formative work was completed (please see lines xxx-yyy).*

19. Line 254 -- The selected TBYs, as opposed to TBYs selected.

*We have made this edit.*

20. Line 309 -- this particular approach

*This edit was completed.*

21. Lines 319-324 -- Strengths and Limitations should be moved to Discussion instead of Conclusions.

*This edit was completed.*

22. Table 1 -- You might consider collapsing some categories as many of your cell sizes are n=1.

*Given the small sample size and that we are including multiple phases in Table 1, we have not collapsed categories.*

23. Table 3 -- Could add some scoring details here.

*As noted elsewhere, we did not develop an overall scoring approach using the pilot data due to the small sample size; that analysis will be done in the larger implementation-effectiveness study. Please see Table 3 for information on scoring.*

24. Figure 1 -- Does not add much, could use the space for something else like table with codebook, example interview questions, or scoring details

*In response to Reviewer #1, we have kept and updated this figure to include the timeframes for each Phase, which is important in providing a visual explanation of the process including where phases overlapped.*

25. Supplemental Info --- Attachment of IRB approval letters to manuscripts typically not necessary. Stating IRB approval in Methods is enough. Could use Supplement for codebook, example interview questions, scoring details and/or saturation table instead.

*This was required by the journal for submission.*

Editor’s Comments: Key Areas Requiring Revision. In preparing your revision, please pay particular attention to the following areas, which were raised by both reviewers, sometimes from different perspectives.

- Clarification of Tool Purpose, Scoring, and Interpretation: Clearly describe how the tool is intended to be used by people with HIV in practice. Explicitly explain how Likert-scale items are scored, whether items are summed or interpreted individually, and whether any cut-points, thresholds, or decision rules are implied. Clarify what constitutes a “suitable” versus “unsuitable” treatment buddy and how scores should inform patient–clinician discussion and counseling.

*See responses to Reviewer 1 (#2, 3, 6) and Reviewer 2 (#7, 8, 23).*

- Methods Transparency and Consistency: Provide additional detail on the development of interview guides, including example questions (ideally in a table or Supplement). Clarify the role and consent of treatment buddies, particularly in the pilot phase, to avoid confusion regarding who used the tool and for what purpose. Address inconsistencies or ambiguities in the study timeline (e.g., interview and pilot phases). More clearly describe the Community Advisory Panel (CAP), including its function, sampling strategy, and how it differs from cognitive interviewing.

*See responses to Reviewer 1 (#2, 3, 6) and Reviewer 2 (#2, 4, 6, 14, 15, 16).*

- Dyadic Framing and Terminology: Reviewer 1 raised concerns about the use of “dyads” without corresponding dyadic analysis, while Reviewer 2 encouraged stronger linkage to dyadic literature. To resolve this tension, we suggest that you: Avoid implying dyadic analysis if none was conducted; and Retain discussion of dyadic concepts at a conceptual or theoretical level in the Discussion, with appropriate caveats.

*See responses to Reviewer 1 (#9) and Reviewer 2 (#3).*

- Results Presentation and Ceiling Effects: Expand discussion of pilot results where ceiling effects were observed, including implications for interpretation, tool sensitivity, and future validation. Consider whether scoring or item distributions should be more explicitly contextualized in the Results and Discussion.

*See responses to Reviewer 1 (#6) and Reviewer 2 (#7, 8, 23).*

- Discussion and Limitations: -Strengthen the Discussion by explicitly addressing: Differences and similarities across clinician, patient, and TBY perspectives; Cultural, relational, and contextual considerations in TBY selection; The small pilot sample size and urban study setting; Planned next steps for validation, including reliability and internal consistency testing. Ensure strengths and limitations are clearly delineated and appropriately placed within the Discussion.

*See responses to Reviewer 1 (#2, 3, 6) and Reviewer 2 (#21).*

- Manuscript Organization and Redundancy: Consolidate repetitive sections in the Introduction, particularly overlapping background paragraphs. Consider whether figures or tables (e.g., Figure 1) add substantive value or could be replaced with content more directly supporting methodological clarity.

*See responses to Reviewer 1 (#10) and Reviewer 2 (#1).*
