# Supplementary material for: “Picking the right person … can make or break the whole deal”: Development of a layperson injector selection tool for administration of home-based long-acting injectable antiretroviral therapies
Source: PLoS One. 2026 Jun 12;21(6):e0351213. doi: 10.1371/journal.pone.0351213 (PMC13262845; doi:10.1371/journal.pone.0351213)
Supplement: Peer review history

# Peer review: Decision Letter 0

**DOI:** 10.1371/journal.pone.0351213.r001

**Authors:** Omar Sued

20 Mar 2026

PONE-D-25-67939

"Picking the right person ... can make or break the whole deal": Development of a layperson injector selection tool for administration of home-based long-acting injectable antiretroviral therapies

PLOS One

Dear Dr. Bourdeau,

Thank you for submitting your manuscript to PLOS ONE. After careful consideration, we feel that it has merit but does not fully meet PLOS ONE’s publication criteria as it currently stands. Therefore, we invite you to submit a revised version of the manuscript that addresses the points raised during the review process.

Please submit your revised manuscript by May 03 2026 11:59PM. If you will need more time than this to complete your revisions, please reply to this message or contact the journal office at plosone@plos.org. When you're ready to submit your revision, log on to https://www.editorialmanager.com/pone/ and select the 'Submissions Needing Revision' folder to locate your manuscript file.

Please include the following items when submitting your revised manuscript:

- A letter that responds to each point raised by the academic editor and reviewer(s). You should upload this letter as a separate file labeled 'Response to Reviewers'.
- A marked-up copy of your manuscript that highlights changes made to the original version. You should upload this as a separate file labeled 'Revised Manuscript with Track Changes'.
- An unmarked version of your revised paper without tracked changes. You should upload this as a separate file labeled 'Manuscript'.

If you would like to make changes to your financial disclosure, please include your updated statement in your cover letter. Guidelines for resubmitting your figure files are available below the reviewer comments at the end of this letter.

If applicable, we recommend that you deposit your laboratory protocols in protocols.io to enhance the reproducibility of your results. Protocols.io assigns your protocol its own identifier (DOI) so that it can be cited independently in the future. For instructions see: https://journals.plos.org/plosone/s/submission-guidelines#loc-laboratory-protocols. Additionally, PLOS ONE offers an option for publishing peer-reviewed Lab Protocol articles, which describe protocols hosted on protocols.io. Read more information on sharing protocols at https://plos.org/protocols?utm_medium=editorial-email&utm_source=authorletters&utm_campaign=protocols.

We look forward to receiving your revised manuscript.

Kind regards,

Omar Sued, MD, PhD

Academic Editor

PLOS One

Journal Requirements:

When submitting your revision, we need you to address these additional requirements.

1. Please ensure that your manuscript meets PLOS ONE's style requirements, including those for file naming. The PLOS ONE style templates can be found at

https://journals.plos.org/plosone/s/file?id=wjVg/PLOSOne_formatting_sample_main_body.pdf and https://journals.plos.org/plosone/s/file?id=ba62/PLOSOne_formatting_sample_title_authors_affiliations.pdf

2. Thank you for stating in your Funding Statement:

[This work was supported by the Centers for Disease Control and Prevention (CDC),

U.S. Department of Health and Human Services (HHS), under grant number

5U01PS005259 awarded to PS. The funder's website is: https://www.cdc.gov.

Collaborators from the funding agency are included as authors of this manuscript.].

Please provide an amended statement that declares *all* the funding or sources of support (whether external or internal to your organization) received during this study, as detailed online in our guide for authors at http://journals.plos.org/plosone/s/submit-now. Please also include the statement “There was no additional external funding received for this study.” in your updated Funding Statement.

Please include your amended Funding Statement within your cover letter. We will change the online submission form on your behalf.

3. Please note that funding information should not appear in any section or other areas of your manuscript. We will only publish funding information present in the Funding Statement section of the online submission form. Please remove any funding-related text from the manuscript.

4. In the online submission form you indicate that your data is not available for proprietary reasons and have provided a contact point for accessing this data. Please note that your current contact point is a co-author on this manuscript. According to our Data Policy, the contact point must not be an author on the manuscript and must be an institutional contact, ideally not an individual. Please revise your data statement to a non-author institutional point of contact, such as a data access or ethics committee, and send this to us via return email. Please also include contact information for the third-party organization, and please include the full citation of where the data can be found.

5. When completing the data availability statement of the submission form, you indicated that you will make your data available on acceptance. We strongly recommend all authors decide on a data sharing plan before acceptance, as the process can be lengthy and hold up publication timelines. Please note that, though access restrictions are acceptable now, your entire data will need to be made freely accessible if your manuscript is accepted for publication. This policy applies to all data except where public deposition would breach compliance with the protocol approved by your research ethics board. If you are unable to adhere to our open data policy, please kindly revise your statement to explain your reasoning and we will seek the editor's input on an exemption. Please be assured that, once you have provided your new statement, the assessment of your exemption will not hold up the peer review process.

6. Please include a separate caption for each figure in your manuscript.

7. Please remove all personal information in the manuscript, ensure that the data shared are in accordance with participant consent, and re-upload a fully anonymized data set.

The following are examples of data that should not be shared:

- Names, nicknames, and initials

- Age more specific than round numbers

- GPS coordinates, physical addresses, IP addresses, email addresses

- Information in small sample sizes (e.g. 40 students from X class in X year at X university)

- Specific dates (e.g. visit dates, interview dates)

- ID numbers

Data that are not directly identifying may also be inappropriate to share, as in combination they can become identifying. For example, data collected from a small group of participants, vulnerable populations, or private groups should not be shared if they involve indirect identifiers (such as sex, ethnicity, location, etc.) that may risk the identification of study participants.

Additional guidance on preparing raw data for publication can be found in our Data Policy (https://journals.plos.org/plosone/s/data-availability#loc-human-research-participant-data-and-other-sensitive-data) and in the following article: http://www.bmj.com/content/340/bmj.c181.long.

8. We note that this manuscript consists of interview transcripts. Can you please confirm that all participants gave consent for interview transcript to be published?

If they DID provide consent for these transcripts to be published, please also confirm that the transcripts do not contain any potentially identifying information (or let us know if the participants consented to having their personal details published and made publicly available). We consider the following details to be identifying information:

- Names, nicknames, and initials

- Age more specific than round numbers

- GPS coordinates, physical addresses, IP addresses, email addresses

- Information in small sample sizes (e.g. 40 students from X class in X year at X university)

- Specific dates (e.g. visit dates, interview dates)

- ID numbers

Or, if the participants DID NOT provide consent for these transcripts to be published:

- Provide a de-identified version of the data or excerpts of interview responses

- Provide information regarding how these transcripts can be accessed by researchers who meet the criteria for access to confidential data, including:

a) the grounds for restriction

b) the name of the ethics committee, Institutional Review Board, or third-party organization that is imposing sharing restrictions on the data

c) a non-author, institutional point of contact that is able to field data access queries, in the interest of maintaining long-term data accessibility.

d) Any relevant data set names, URLs, DOIs, etc. that an independent researcher would need in order to request your minimal data set.

For further information on sharing data that contains sensitive participant information, please see: https://journals.plos.org/plosone/s/data-availability#loc-human-research-participant-data-and-other-sensitive-data

If there are ethical, legal, or third-party restrictions upon your dataset, you must provide all of the following details (https://journals.plos.org/plosone/s/data-availability#loc-acceptable-data-access-restrictions):

1. A complete description of the dataset

2. The nature of the restrictions upon the data (ethical, legal, or owned by a third party) and the reasoning behind them

3. The full name of the body imposing the restrictions upon your dataset (ethics committee, institution, data access committee, etc)

4. If the data are owned by a third party, confirmation of whether the authors received any special privileges in accessing the data that other researchers would not have

5. Direct, non-author contact information (preferably email) for the body imposing the restrictions upon the data, to which data access requests can be sent

9. Please include captions for your Supporting Information files at the end of your manuscript, and update any in-text citations to match accordingly. Please see our Supporting Information guidelines for more information: http://journals.plos.org/plosone/s/supporting-information.

If the reviewer comments include a recommendation to cite specific previously published works, please review and evaluate these publications to determine whether they are relevant and should be cited. There is no requirement to cite these works unless the editor has indicated otherwise.

Additional Editor Comments:

Dear Dr. Bourdeau and colleagues,

Thank you for submitting your manuscript entitled “‘Picking the right person … can make or break the whole deal’: Development of a layperson injector selection tool for administration of home-based long-acting injectable antiretroviral therapies” to PLOS ONE, and for your patience during the review process.

After careful consideration of the manuscript and the two external peer reviews, I am writing to inform you that the editorial decision is Major Revision.

Both reviewers found the study to be well conducted, methodologically sound, and highly relevant to the implementation of long-acting injectable ART, and they recognized the importance of the topic and the strength of the stakeholder-engaged, multi-phase tool development process. However, both reviewers also raised substantive concerns that must be addressed before the manuscript can be considered for publication. These issues primarily relate to clarity, transparency, and interpretation, rather than the overall validity of the work.

Rationale for Major Revision

Although Reviewer 2 characterizes the required changes as “minor,” the reviewers’ collective comments indicate the need for substantial clarification and restructuring across multiple sections of the manuscript, particularly regarding:

The methods and analytic framing

The purpose, scoring, and interpretation of the selection tool

The practical and clinical application of results

The limitations and generalizability of the findings

Addressing these points will require careful revision of the Methods, Results, and Discussion sections, as well as improved alignment across sections. For this reason, a Major Revision decision is appropriate.

Key Areas Requiring Revision

In preparing your revision, please pay particular attention to the following areas, which were raised by both reviewers, sometimes from different perspectives:

1) Clarification of Tool Purpose, Scoring, and Interpretation

Clearly describe how the tool is intended to be used by people with HIV in practice.

Explicitly explain how Likert-scale items are scored, whether items are summed or interpreted individually, and whether any cut-points, thresholds, or decision rules are implied.

Clarify what constitutes a “suitable” versus “unsuitable” treatment buddy and how scores should inform patient–clinician discussion and counseling.

2) Methods Transparency and Consistency

Provide additional detail on the development of interview guides, including example questions (ideally in a table or Supplement).

Clarify the role and consent of treatment buddies, particularly in the pilot phase, to avoid confusion regarding who used the tool and for what purpose.

Address inconsistencies or ambiguities in the study timeline (e.g., interview and pilot phases).

More clearly describe the Community Advisory Panel (CAP), including its function, sampling strategy, and how it differs from cognitive interviewing.

3) Dyadic Framing and Terminology

Reviewer 1 raised concerns about the use of “dyads” without corresponding dyadic analysis, while Reviewer 2 encouraged stronger linkage to dyadic literature.

To resolve this tension, we suggest that you:

Avoid implying dyadic analysis if none was conducted; and

Retain discussion of dyadic concepts at a conceptual or theoretical level in the Discussion, with appropriate caveats.

4) Results Presentation and Ceiling Effects

Expand discussion of pilot results where ceiling effects were observed, including implications for interpretation, tool sensitivity, and future validation.

Consider whether scoring or item distributions should be more explicitly contextualized in the Results and Discussion.

5) Discussion and Limitations

-Strengthen the Discussion by explicitly addressing:

Differences and similarities across clinician, patient, and TBY perspectives;

Cultural, relational, and contextual considerations in TBY selection;

The small pilot sample size and urban study setting;

Planned next steps for validation, including reliability and internal consistency testing.

-Ensure strengths and limitations are clearly delineated and appropriately placed within the Discussion.

6) Manuscript Organization and Redundancy

Consolidate repetitive sections in the Introduction, particularly overlapping background paragraphs.

Consider whether figures or tables (e.g., Figure 1) add substantive value or could be replaced with content more directly supporting methodological clarity.

7) Reviewer Tensions

We note some differences in emphasis between the two reviews (e.g., degree of revision required, dyadic framing). These are not contradictory but reflect different perspectives. You are not expected to “choose” between reviewers; rather, please address the underlying concern raised by each, clearly explaining how revisions resolve them. Where you decide not to implement a suggestion, please provide a concise justification in your response letter.

Please submit:

A revised manuscript with tracked changes or clearly indicated revisions; and

A detailed, point-by-point response to each reviewer comment.

For your convenience, both reviewer reports are appended below.

We appreciate the importance and promise of this work and encourage you to revise and resubmit. We look forward to reviewing a revised version that addresses the issues raised and strengthens the manuscript’s clarity, interpretability, and applicability.

Sincerely,

Omar Sued

Academic Editor

PLOS ONE

[Note: HTML markup is below. Please do not edit.]

Reviewers' comments:

Reviewer's Responses to Questions

**Comments to the Author**

1. Is the manuscript technically sound, and do the data support the conclusions?

The manuscript must describe a technically sound piece of scientific research with data that supports the conclusions. Experiments must have been conducted rigorously, with appropriate controls, replication, and sample sizes. The conclusions must be drawn appropriately based on the data presented.

Reviewer #1: Yes

Reviewer #2: Yes

**********

2. Has the statistical analysis been performed appropriately and rigorously?

Reviewer #1: Yes

Reviewer #2: N/A

**********

3. Have the authors made all data underlying the findings in their manuscript fully available?

The PLOS Data policy requires authors to make all data underlying the findings described in their manuscript fully available without restriction, with rare exception (please refer to the Data Availability Statement in the manuscript PDF file). The data should be provided as part of the manuscript or its supporting information, or deposited to a public repository. For example, in addition to summary statistics, the data points behind means, medians and variance measures should be available. If there are restrictions on publicly sharing data—e.g. participant privacy or use of data from a third party—those must be specified.

Reviewer #1: Yes

Reviewer #2: Yes

**********

4. Is the manuscript presented in an intelligible fashion and written in standard English?

PLOS ONE does not copyedit accepted manuscripts, so the language in submitted articles must be clear, correct, and unambiguous. Any typographical or grammatical errors should be corrected at revision, so please note any specific errors here.

Reviewer #1: Yes

Reviewer #2: Yes

**********

5. Review Comments to the Author

Please use the space provided to explain your answers to the questions above. You may also include additional comments for the author, including concerns about dual publication, research ethics, or publication ethics. (Please upload your review as an attachment if it exceeds 20,000 characters)

Reviewer #1: Overall thoughts: This is a well-conducted, methodologically sound study addressing an important gap in LAI-ART implementation. The multi-phase, stakeholder-engaged tool development process is exemplary, and findings have direct practical implications for expanding home-based HIV care. With making minor revisions detailed here, this manuscript will make a strong contribution to PLOS ONE.

Major issues:

• Authors should be more explicit in their discussion of limitations of the study and comment on the small sample size of this pilot (n=7) in addition to the fact that all participants are from urban settings (San Francisco Bay Area clinics), as this may affect real world performance

• Furthermore, authors are encouraged to include preliminary internal consistency measures from the pilot data and outline validation plans for future work to address the lack of reliability analysis from the study.

• Even though the study generates important data on the subject, information on contextualizing results is missing. Authors should include a section on interpretation of scores and clinical application of results i.e., what exactly does ‘suitable’ or ‘unsuitable’ TBY denote? How should clinicians apply the scores as part of counselling?

Minor issues:

• Authors state that semi-structured interviews were conducted between June 2024 and April 2025 (lines 103-105), and pilot testing (phase 3, line 136-137) from June to September 2025? Kindly align timeline

• Kindly clarify the sampling strategy employed in phase 4 (Community Advisory Panel) and report participation rate

• Further discussion is required for all scores greater or equal to 3 under the ceiling effects under the results section

• Phase 2: Drafting the Tool (results, lines 232-249) could be clearer if presented in chronological order, consider revising.

• Kindly specify the ‘missing’ on Table 1, is it clinicians or TBY?

• Although the discussion is well-written, it can be further enhanced by addressing cultural considerations in TBY selection and strengthening parallels to dyadic HIV/health literature with specific examples

• Lines 40-51 and 53-63 appear repetitive, consider consolidating.

Reviewer #2: Thank you for the opportunity to review this manuscript on the development of a treatment buddy (TBY) selection tool for patients receiving long-acting injectable antiretroviral therapy (LAI-ART). This manuscript addresses access, confidentiality and comfort around treatment options for PLWHA, with the goal of having more PLWHA with undetectable status'. While this manuscript adds to the literature about LAI-ART and alternative delivery models, there are several areas of concern which I detail below:

Major:

Introduction --

lines 53-63 --- seems repetitive and doesn't add much, therefore could be woven into paragraphs 1-2 (lines 29-52). It would be better served to outline any prior buddy selection tools that have been used in this population or others. If this is a novel idea, there should be more background as to why the authors thought this would work in this population.

Methods --

lines 102-110 -- How were the guides developed? Could you provide example questions in a table in the supplement or if you feel important, into the main paper? Were there separate guides for clinicians, patients and TBYs? What topics were asked about (I see briefly on lines 88-89, but that is too brief and should be probably moved to around lines 102)?

lines 96-101 -- Seems like most patients were paired with their TBYs and you mention dyads later, but no mention of dyadic analysis in your methodology. If you did do dyadic analysis, you should explain more in the Methods. If you did not, I would probably drop mentions of dyads (lines 164 and 289).

line 127 -- there needs to be a sentence or two making it clear that the tool is meant to be used by PLHWA to identify TBY because in the pilot test, TBYs were also consented for some reason and it was a little confusing.

lines 141-143 -- the consenting of TBYs for the pilot test is confusing. Perhaps they were consented for another process that is not mentioned in the manuscript. For that reason, either clarify why that needed to happen (what role TBYs played in the pilot test -- did the PLWHA verbally ask the TBYs questions from the selection tool?) or remove.

lines 148-149 -- More needs to be described about the invitation of Phase 1 participants to the CAP and why that decision was made (recruitment difficulties?). It seems like the CAP served as a group cognitive interview to refine the tool more that an advisory panel. An advisory panel seems like an opportunity to get other stakeholders involved (experts, patient advocacy groups, etc.). Methodologically, the CAP should probably be described more as a cognitive interview vs. panel.

Results --

Line 249 -- No mention of how the tool is scored after revision. You mention Likert scales, but scoring and decisions about any reverse coding, etc. need to be described. And how does the scoring help the PLWHA decide to select that person as a TBY or not? That is really important to include.

Lines 272-277 -- Another opportunity to discuss scoring here. And how do you envision tool use in real-world practice (i.e., does the clinic provide the tool to PLWHA, do they find it online themselves)?

Discussion --

Lines 279-300 -- There needs to be expansion on the similarities and differences between interview findings from clinicians and patients and TBYs, any gaps, what might the study find on expanded testing of the selection tool and how might they envision the rollout of this delivery model?

Minor:

Introduction --

line 34 -- there is no mention of side effects (or lack of) of LAI-ART along with effectiveness and acceptability.

line 35 -- you might provide a better term than specialized personnel, as it is not clear what is meant (do you mean prescribers?) It begs the question, if specialized personnel are needed in the clinic, how can the treatment be delivered by TBYs?

lines 44-45 --- decentralized drug delivery models as opposed to injection models

Methods --

lines 84-86 -- is it important to name the sites? May de-identify since there are small participant numbers.

line 111 -- thematically analyzed

line 116 -- consider adding codebook with themes, subthemes and descriptions to a table in main text or supplement

line 119 -- consider adding saturation table by theme/subtheme into supplement

Results --

line 233 -- study team or coding team members as opposed to analysts

line 234 -- Is "1-year" commitment important? This is the only time it is mentioned and is very specific. Why not just commitment to injection schedule with no time frame?

Line 254 -- The selected TBYs, as opposed to TBYs selected.

Line 309 -- this particular approach

Lines 319-324 -- Strengths and Limitations should be moved to Discussion instead of Conclusions.

Table 1 -- You might consider collapsing some categories as many of your cell sizes are n=1.

Table 3 -- Could add some scoring details here.

Figure 1 -- Does not add much, could use the space for something else like table with codebook, example interview questions, or scoring details

Supplemental Info --- Attachment of IRB approval letters to manuscripts typically not necessary. Stating IRB approval in Methods is enough. Could use Supplement for codebook, example interview questions, scoring details and/or saturation table instead.

**********

6. PLOS authors have the option to publish the peer review history of their article (what does this mean?). If published, this will include your full peer review and any attached files.

If you choose “no”, your identity will remain anonymous but your review may still be made public.

**Do you want your identity to be public for this peer review?**  For information about this choice, including consent withdrawal, please see our Privacy Policy.

Reviewer #1: No

Reviewer #2: No

**********

[NOTE: If reviewer comments were submitted as an attachment file, they will be attached to this email and accessible via the submission site. Please log into your account, locate the manuscript record, and check for the action link "View Attachments". If this link does not appear, there are no attachment files.]

To ensure your figures meet our technical requirements, please review our figure guidelines: https://journals.plos.org/plosone/s/figures

You may also use PLOS’s free figure tool, NAAS, to help you prepare publication quality figures: https://journals.plos.org/plosone/s/figures#loc-tools-for-figure-preparation.

NAAS will assess whether your figures meet our technical requirements by comparing each figure against our figure specifications.

# Author comment: Author response to Decision Letter 1

**DOI:** 10.1371/journal.pone.0351213.r002

21 May 2026

Response to Reviewers

Reviewer #1: Overall thoughts: This is a well-conducted, methodologically sound study addressing an important gap in LAI-ART implementation. The multi-phase, stakeholder-engaged tool development process is exemplary, and findings have direct practical implications for expanding home-based HIV care. With making minor revisions detailed here, this manuscript will make a strong contribution to PLOS ONE.

We thank the Reviewer for their helpful comments.

Major issues:

1. Authors should be more explicit in their discussion of limitations of the study and comment on the small sample size of this pilot (n=7) in addition to the fact that all participants are from urban settings (San Francisco Bay Area clinics), as this may affect real world performance

We have added further details in the discussion section about the limitations of the study, specifically a paragraph at the end of the Discussion section (lines 347 to 368).

2. Furthermore, authors are encouraged to include preliminary internal consistency measures from the pilot data and outline validation plans for future work to address the lack of reliability analysis from the study.

This is an important point that we have not yet been able to test with our limited dataset. We have added additional details in the discussion about this limitation and plans for future exploration of these points (see paragraph at the end of the discussion section).

3. Even though the study generates important data on the subject, information on contextualizing results is missing. Authors should include a section on interpretation of scores and clinical application of results i.e., what exactly does ‘suitable’ or ‘unsuitable’ TBY denote? How should clinicians apply the scores as part of counselling?

We have added sentences at the end of the first two discussion paragraphs on the applications of this tool, such as those mentioned, that we hope to determine through the larger study.

Minor issues:

4. Authors state that semi-structured interviews were conducted between June 2024 and April 2025 (lines 103-105), and pilot testing (phase 3, line 136-137) from June to September 2025? Kindly align timeline

The timelines reflect different phases of the study. The qualitative interviews (Phase 1) were conducted between June 2024 and April 2025, and findings from these interviews informed the development of the tool. Pilot testing (Phase 3) was conducted afterward, from June to September 2025. Figure 1 has been modified to include the dates for each phase, some of which overlap.

5. Kindly clarify the sampling strategy employed in phase 4 (Community Advisory Panel) and report participation rate

We have updated the Phase 4 description (first sentence) to note that all Phase 3 participants were invited; we used a convenience sample.

6. Further discussion is required for all scores greater or equal to 3 under the ceiling effects under the results section

We have clarified this limitation in the discussion section (the last paragraph before the Conclusions).

7. Phase 2: Drafting the Tool (results, lines 232-249) could be clearer if presented in chronological order, consider revising.

The Phase 2 description has been edited to convey the order in which modifications were made in the tool’s initial drafts, throughout the entire section.

8. Kindly specify the ‘missing’ on Table 1, is it clinicians or TBY?

We had noted by the (†) symbol that demographics were collected only from the PWH/TBY participants. We have added the (†) symbol to each of the demographic categories to denote that the numbers and percentages provided pertain only to PWH/TBY and not to clinicians.

9. Although the discussion is well-written, it can be further enhanced by addressing cultural considerations in TBY selection and strengthening parallels to dyadic HIV/health literature with specific examples

We have added more context to the discussion as suggested, with greater anchoring of our findings to the dyadic health literature (with examples).

10. Lines 40-51 and 53-63 appear repetitive, consider consolidating.

Those two paragraphs have been consolidated.

Reviewer #2: Thank you for the opportunity to review this manuscript on the development of a treatment buddy (TBY) selection tool for patients receiving long-acting injectable antiretroviral therapy (LAI-ART). This manuscript addresses access, confidentiality and comfort around treatment options for PLWHA, with the goal of having more PLWHA with undetectable status'. While this manuscript adds to the literature about LAI-ART and alternative delivery models, there are several areas of concern which I detail below:

Thank you for this review and suggestions for further improvement.

Major:

Introduction --

1. lines 53-63 --- seems repetitive and doesn't add much, therefore could be woven into paragraphs 1-2 (lines 29-52). It would be better served to outline any prior buddy selection tools that have been used in this population or others. If this is a novel idea, there should be more background as to why the authors thought this would work in this population.

We have consolidated the two paragraphs into one.

Methods --

2. lines 102-110 -- How were the guides developed? Could you provide example questions in a table in the supplement or if you feel important, into the main paper? Were there separate guides for clinicians, patients and TBYs? What topics were asked about (I see briefly on lines 88-89, but that is too brief and should be probably moved to around lines 102)?

We have added a paragraph describing the topics and questions on the interview guides for each of the three categories of participants (paragraph 4 of this section).

3. lines 96-101 -- Seems like most patients were paired with their TBYs and you mention dyads later, but no mention of dyadic analysis in your methodology. If you did do dyadic analysis, you should explain more in the Methods. If you did not, I would probably drop mentions of dyads (lines 164 and 289).

While participants were recruited as PWH-TBY pairs, we did not conduct a formal dyadic analysis. As noted in the Methods, interviews were analyzed separately by participant group (clinicians, PWH, and TBYs) to capture distinct perspectives. We have added clarifying language to make this explicit (starting on line 119) and revised references to “dyads” to “duos” to better reflect the study design.

4. line 127 -- there needs to be a sentence or two making it clear that the tool is meant to be used by PLHWA to identify TBY because in the pilot test, TBYs were also consented for some reason and it was a little confusing.

The order of activities was changed so that the PWH completed the TBY Selection Tool before contacting the TBY for participation (end of first paragraph).

5. lines 141-143 -- the consenting of TBYs for the pilot test is confusing. Perhaps they were consented for another process that is not mentioned in the manuscript. For that reason, either clarify why that needed to happen (what role TBYs played in the pilot test -- did the PLWHA verbally ask the TBYs questions from the selection tool?) or remove.

The pilot study collected data from both members of the duos; however, no data collected from the TBY is presented in this paper. Mention of consent and data collection from the TBY has been removed for clarity.

6. lines 148-149 -- More needs to be described about the invitation of Phase 1 participants to the CAP and why that decision was made (recruitment difficulties?). It seems like the CAP served as a group cognitive interview to refine the tool more that an advisory panel. An advisory panel seems like an opportunity to get other stakeholders involved (experts, patient advocacy groups, etc.). Methodologically, the CAP should probably be described more as a cognitive interview vs. panel.

While this session with the CAP was used to gather members’ input on the TBY selection tool, we have and continue to involve the CAP in various aspects of the research. We had few difficulties in recruiting CAP members because they were clinicians from partnering clinics and PWH/TBYs who were interested in this topic.

Results --

7. Line 249 -- No mention of how the tool is scored after revision. You mention Likert scales, but scoring and decisions about any reverse coding, etc. need to be described. And how does the scoring help the PLWHA decide to select that person as a TBY or not? That is really important to include.

This is the intended function of the TBY Selection Tool and will be the analytical goal of the full study. Additional details on how we addressed the small pilot dataset are provided (see second paragraph of Phase 3 [Results]).

8. Lines 272-277 -- Another opportunity to discuss scoring here. And how do you envision tool use in real-world practice (i.e., does the clinic provide the tool to PLWHA, do they find it online themselves)?

Please see comment above.

Discussion --

9. Lines 279-300 -- There needs to be expansion on the similarities and differences between interview findings from clinicians and patients and TBYs, any gaps, what might the study find on expanded testing of the selection tool and how might they envision the rollout of this delivery model?

We have expanded the Discussion section (second paragraph) to more explicitly synthesize key similarities and differences across clinician, PWH, and TBY perspectives, including areas of alignment and divergence.

Minor:

Introduction --

10. line 34 -- there is no mention of side effects (or lack of) of LAI-ART along with effectiveness and acceptability.

While we concur that side effects play a role in uptake of injectable ART, our focus here is limited to implementation issues for those interested in and tolerant of injectable ART. A shift in the delivery model will not remedy side effects that are sufficiently problematic as to impact their use.

11. line 35 -- you might provide a better term than specialized personnel, as it is not clear what is meant (do you mean prescribers?) It begs the question, if specialized personnel are needed in the clinic, how can the treatment be delivered by TBYs?

This term has been correctly flagged as inaccurate. We have changed it to ‘dedicated’ which better reflects the impact of injectable programs on workflow and staffing.

12. lines 44-45 --- decentralized drug delivery models as opposed to injection models

We concur and have made the edit.

Methods --

13. lines 84-86 -- is it important to name the sites? May de-identify since there are small participant numbers.

We have removed the clinic names.

14. line 111 -- thematically analyzed

We have clarified the analytic approach by specifying that a combination of inductive and deductive analysis was used.

15. line 116 -- consider adding codebook with themes, subthemes and descriptions to a table in main text or supplement

The key themes are listed in the first sentence for each participant group; the analytic approach is described in the Methods for Phase 1 in paragraph two.

16. line 119 -- consider adding saturation table by theme/subtheme into supplement

We clarified that themes were developed and refined iteratively until saturation was reached.

Results --

17. line 233 -- study team or coding team members as opposed to analysts

We have made this edit.

18. line 234 -- Is "1-year" commitment important? This is the only time it is mentioned and is very specific. Why not just commitment to injection schedule with no time frame?

We have clarified this requirement as it is the length of the implementation-effectiveness study for which this formative work was completed (please see lines xxx-yyy).

19. Line 254 -- The selected TBYs, as opposed to TBYs selected.

We have made this edit.

20. Line 309 -- this particular approach

This edit was completed.

21. Lines 319-324 -- Strengths and Limitations should be moved to Discussion instead of Conclusions.

This edit was completed.

22. Table 1 -- You might consider collapsing some categories as many of your cell sizes are n=1.

Given the small sample size and that we are including multiple phases in Table 1, we have not collapsed categories.

23. Table 3 -- Could add some scoring details here.

As noted elsewhere, we did not develop an overall scoring approach using the pilot data due to the small sample size; that analysis will be done in the larger implementation-effectiveness study. Please see Table 3 for information on scoring.

24. Figure 1 -- Does not add much, could use the space for something else like table with codebook, example interview questions, or scoring details

In response to Reviewer #1, we have kept and updated this figure to include the timeframes for each Phase, which is important in providing a visual explanation of the process including where phases overlapped.

25. Supplemental Info --- Attachment of IRB approval letters to manuscripts typically not necessary. Stating IRB approval in Methods is enough. Could use Supplement for codebook, example interview questions, scoring details and/or saturation table instead.

This was required by the journal for submission.

Editor’s Comments: Key Areas Requiring Revision. In preparing your revision, please pay particular attention to the following areas, which were raised by both reviewers, sometimes from different perspectives.

• Clarification of Tool Purpose, Scoring, and Interpretation: Clearly describe how the tool is intended to be used by people with HIV in practice. Explicitly explain how Likert-scale items are scored, whether items are summed or interpreted individually, and whether any cut-points, thresholds, or decision rules are implied. Clarify what constitutes a “suitable” versus “unsuitable” treatment buddy and how scores should inform patient–clinician discussion and counseling.

See responses to Reviewer 1 (#2, 3, 6) and Reviewer 2 (#7, 8, 23).

• Methods Transparency and Consistency: Provide additional detail on the development of interview guides, including example questions (ideally in a table or Supplement). Clarify the role and consent of treatment buddies, particularly in the pilot phase, to avoid confusion regarding who used the tool and for what purpose. Address inconsistencies or ambiguities in the study timeline (e.g., interview and pilot phases). More clearly describe the Community Advisory Panel (CAP), including its function, sampling strategy, and how it differs from cognitive interviewing.

See responses to Reviewer 1 (#2, 3, 6) and Reviewer 2 (#2, 4, 6, 14, 15, 16).

• Dyadic Framing and Terminology: Reviewer 1 raised concerns about the use of “dyads” without corresponding dyadic analysis, while Reviewer 2 encouraged stronger linkage to dyadic literature. To resolve this tension, we suggest that you: Avoid implying dyadic analysis if none was conducted; and Retain discussion of dyadic concepts at a conceptual or theoretical level in the Discussion, with appropriate caveats.

See responses to Reviewer 1 (#9) and Reviewer 2 (#3).

• Results Presentation and Ceiling Effects: Expand discussion of pilot results where ceiling effects were observed, including implications for interpretation, tool sensitivity, and future validation. Consider whether scoring or item distributions should be more explicitly contextualized in the Results and Discussion.

See responses to Reviewer 1 (#6) and Reviewer 2 (#7, 8, 23).

• Discussion and Limitations: -Strengthen the Discussion by explicitly addressing: Differences and similarities across clinician, patient, and TBY perspectives; Cultural, relational, and contextual considerations in TBY selection; The small pilot sample size and urban study setting; Planned next steps for validation, including reliability and internal consistency testing. Ensure strengths and limitations are clearly delineated and appropriately placed within the Discussion.

See responses to Reviewer 1 (#2, 3, 6) and Reviewer 2 (#21).

• Manuscript Organization and Redundancy: Consolidate repetitive sections in the Introduction, particularly overlapping background paragraphs. Consider whether figures or tables (e.g., Figure 1) add substantive value or could be replaced with content more directly supporting methodological clarity.

See responses to Reviewer 1 (#10) and Reviewer 2 (#1).

Attachment Submitted filename: Response to Reviewers.docx

# Editor report: Decision Letter 1

**DOI:** 10.1371/journal.pone.0351213.r003

**Authors:** Omar Sued

26 May 2026

"Picking the right person ... can make or break the whole deal": Development of a layperson injector selection tool for administration of home-based long-acting injectable antiretroviral therapies

PONE-D-25-67939R1

Dear Dr. Bourdeau,

We’re pleased to inform you that your manuscript has been judged scientifically suitable for publication and will be formally accepted for publication once it meets all outstanding technical requirements.

Within one week, you’ll receive an e-mail detailing the required amendments. When these have been addressed, you’ll receive a formal acceptance letter and your manuscript will be scheduled for publication.

An invoice will be generated when your article is formally accepted. Please note, if your institution has a publishing partnership with PLOS and your article meets the relevant criteria, all or part of your publication costs will be covered. Please make sure your user information is up-to-date by logging into Editorial Manager at Editorial Manager® and clicking the ‘Update My Information' link at the top of the page. For questions related to billing, please contact billing support.

If your institution or institutions have a press office, please notify them about your upcoming paper to help maximize its impact. If they’ll be preparing press materials, please inform our press team as soon as possible -- no later than 48 hours after receiving the formal acceptance. Your manuscript will remain under strict press embargo until 2 pm Eastern Time on the date of publication. For more information, please contact onepress@plos.org.

Kind regards,

Omar Sued, MD, PhD

Academic Editor

PLOS One

Additional Editor Comments (optional):

Reviewers' comments:

# Editor report: Acceptance letter

**DOI:** 10.1371/journal.pone.0351213.r004

**Authors:** Omar Sued

PONE-D-25-67939R1

PLOS One

Dear Dr. Bourdeau,

I'm pleased to inform you that your manuscript has been deemed suitable for publication in PLOS One. Congratulations! Your manuscript is now being handed over to our production team.

At this stage, our production department will prepare your paper for publication. This includes ensuring the following:

* All references, tables, and figures are properly cited

* All relevant supporting information is included in the manuscript submission,

* There are no issues that prevent the paper from being properly typeset

You will receive further instructions from the production team, including instructions on how to review your proof when it is ready. Please keep in mind that we are working through a large volume of accepted articles, so please give us a few days to review your paper and let you know the next and final steps.

Lastly, if your institution or institutions have a press office, please let them know about your upcoming paper now to help maximize its impact. If they'll be preparing press materials, please inform our press team within the next 48 hours. Your manuscript will remain under strict press embargo until 2 pm Eastern Time on the date of publication. For more information, please contact onepress@plos.org.

You will receive an invoice from PLOS for your publication fee after your manuscript has reached the completed accept phase. If you receive an email requesting payment before acceptance or for any other service, this may be a phishing scheme. Learn how to identify phishing emails and protect your accounts at https://explore.plos.org/phishing.

If we can help with anything else, please email us at customercare@plos.org.

Thank you for submitting your work to PLOS ONE and supporting open access.

Kind regards,

PLOS ONE Editorial Office Staff

on behalf of

Dr. Omar Sued

Academic Editor

PLOS One
